# 

**Published:** 1874-12

**Authors:** 


### OUR BOUND VOLUME.

The Twenty-first Volume of Hall's Journal of Health (for the year 1874), 500 pages, *neatly bound in Cloth*, is now ready for delivery.

Owing to the unexpected demand for copies, and the fact that the plates were not preserved, we are unable to reproduce the work, and we have a limited number only at our disposal. Those who would secure a copy should order without delay. Price $2, postpaid. E. H. Gibbs, 84 Broadway, New York.

